# Perception of Falls and Confidence in Self-Management of Falls among Older Adults

**DOI:** 10.3390/ijerph16245054

**Published:** 2019-12-11

**Authors:** Qiwei Li, Elias Mpofu, Cheng Yin, Keith W. Turner

**Affiliations:** 1 Department of Rehabilitation and Health Services, University of North Texas, Denton, TX 76201, USA; Elias.Mpofu@unt.edu (E.M.); Chengyinunt@gmail.com (C.Y.); Keith.Turner@unt.edu (K.W.T.); 2 Clinical and Rehabilitation Sciences, University of Sydney, Lidcombe 2141, Australia; 3 Educational Psychology and Inclusive Education, University of Johannesburg, Johannesburg 2550, South Africa

**Keywords:** fall management, fear of falls, older adults, confidence, predictors

## Abstract

**Objectives:** Fall preventive programs aim to reduce risks for mortality from fall-related injuries among older adults. However, the covariation between personal perceptions of falls and factors and confidence of self-management in falls (CSMoF) is still under-studied despite its importance to fall prevention. We aimed to investigate the relative contribution of CSMoF in relation to fall risk self-perceptions while controlling for demographics and self-reported health and functioning. **Method:** Participants were 691 older adults recruited from Area Agency on Aging at Arlington, Texas (females = 76.1%, mean age = 76.23, *SD* = 6.44, with chronic condition = 79.5%). They completed measures of physical functioning, CSMoF, fall risk perceptions and fear of falls. *Results:* Regression analyses indicated that fear of fall was the most predictive factor of CSMoF among older persons, accounting for about 25% of the variance. Physical function measures of age, chronic illnesses of metabolism, sensory impairment, and health status were also significant predictors of the CSMoF, but to a lesser extent than fear of falls and fall perceptions. The interaction of perception of falls and fall experience attenuated CSMoF, with physical functioning limitations. **Conclusion:** The joint effects of perception of falls and fear of falls likely explain CSMoF among older adults more than physical functional indicators. Fall prevention programs for older adults should prioritize to address modifiable subjective factors of fall perceptions, fear of falls, and CSMoF across health and functioning statuses.

## 1. Introduction

Approximately one-third of older adults fall each year. Falls are likely to be fatal and costly when they happen to adults over 65 years of age [1] Fall-related mortality rate for American older adults has increased from 43 in 2005 to 62 per million in 2016 [1,2], and fall-related medical service costs are projected to surpass $50 billion by 2020 as baby boomers are passing the 65th year milestone [2]. Evidence shows an estimated 90% of hip fractures result from falls, leading to limited personal mobility [3]. About 2.8 million older adults utilize emergency services each year due to unintentional falls, and over eight million older patients are hospitalized because of fall-related injuries [1,4]. Due to the increased health care expenditures and fall-related health conditions, fall prevention has become a focus of services targeting the aging population.

The following physical function statuses are associated with risk factors of falls among older adults: (a) lower limb weakness; (b) balance deficit; (c) gait abnormalities; (d) visual impairment; (e) mobility limitation; (f) cognitive impairment; (g) impaired functional status; (h) postural hypotension and other chronic conditions; and (i) fear of falling [5,6,7]. Besides, chronic conditions, medications for treating chronic diseases among the aging population may also increase risks of falls through polypharmacy [8,9,10]. As a result, most of the fall prevention programs aim to increase physical strengths such as gait, balance, and functional training. The major interventions include physical exercises and activities enhancing flexibilities [11,12,13].

**Fear of falls research**. Fear of falls is higher among older adults with fall experience as in “post-fall syndrome” [14,15]. The fear of falls could restrict older adults’ confidence in performing activities of daily living, confining the older adults at home, and causing social isolation [16,17]. Fall prevention programs commonly measure and evaluate balance confidence, fall efficacy, and fall management separately. For instance, the *Activities-specific Balance Confidence* (ABC) [18] and the *Falls Efficacy Scale-International* (FES-I) [19] scales are widely used scales to assess the extent to which an older person has concerns about falls when performing given tasks. Conventionally, fall management includes broader factors regarding falls, involves knowledge of fall risks, fall preventive strategies, the relationship between chronic disease and falls, and skills to manage fall when falls happen [20]. For that reason, confidence in self-management of falls (CSMoF) implies knowledge about fall prevention as well as the confidence to achieve target activities without falls.

**Confidence in self-management of falls (CSMoF)**. Confidence in self-management of falls (CSMoF) is protective of fall risks when facing falls risks, despite knowing how to perform risk-free behaviors [21]. This gap between fall risk perception or prevention knowledge and CSMoF may explain both the occurrence and fear of falls. To our best knowledge, previous studies have seldom focused on CSMoF. However, knowing the factors associated with the prediction of CSMoF may contribute to better fall prevention outcomes [22].

Conventionally, the fear of falls is predicated upon one’s physical, psychological, and functional levels. However, the contribution of cognition-related personal factors is underestimated [22]. Studies showed that fear of falls, fall prevention efficacy, and CSMoF are associated with self-rated health [23], fall history [24] and female gender [25], among other demographic variables [26]. Environmental factors such as high social support and living in accessible neighborhoods also contributed to fear of fall mitigation and CSMoF [25,27].

We sought to address two research aims: (a) comparatively predict the CSMoF from sociodemographic factors, health conditions, and perception of falls; and (b) construct an integrated fall mitigation model with older adults based on their self-perception cognitions about risk for falls. Our specific research questions were: (1) To what extent do physical functioning and demographics factors explain the variance in older adults’ subjective perception of falls and their confidence in self-management of falls? (2) What is the relative contribution of subjective perception of fall risk and fear of falls in predicting confidence in self-management of falls?

## 2. Methods

### 2.1. Design and Procedures

This study applied quantitative descriptive research. The Institutional Review Board (IRB) was approved by a public university located in the north Texas area (UNT IRB 17-109). Participants individually consented to the study.

### 2.2. Participants and Setting

We collected the data from a fall prevention program (A Matter of Balance; AMOB) run by the Area Agency on Aging at Arlington, Texas. The AMOB provides physical exercises to increase muscle strength and the Confidence in Self-Management of Falls [28,29]. AMOB provides specific sessions to manage the irrational concerns about falling (Session 5: Managing concerns about falls) and to recognize the fall risks in both home and community levels (Session 7: Identify risk of falls at homes).

Participants 65 and over were eligible for this study. We surveyed a total of 691 aged from 65 to 97 (*M* = 76.23; females, *n* = 526, 76.1%). By ethnicity, the sample comprised European Americans (*n* = 654; 94.7%), followed by Asian Americans (*n* = 20, 2.9%), African Americans (*n* = 10, 1.4%), American Indian or Alaska Native (*n* = 4, 0.6%), and Native Hawaiian or other Pacific Native (*n* = 3, 0.4%). Over half (*n* = 400, 57.9%) of the participants were married, 190 participants were widowed (27.5%), 70 (10.1%) were divorced, and 31 (4.5%) reported other status. Four hundred sixty-two (66.9%) participants were not living alone by the time of registration. Approximately 60% of participants held a college degree and above (*n* = 398), 220 (32%) of participants had some college-level studies or vocational school, and 73 (11%) participants were high school graduates and lower. Table 1 demonstrates the demographic characteristics of the participants.

### 2.3. Measures

**Demographic Variables**. Participants self-reported their demographic characteristics which included age, whether living alone (*yes* or *no*), sex (*male* or *female*), Hispanic (*yes* or *no*), ethnicity (1 = *American Indian or Alaska native*; 2 = *Asian American*; 3 = *Black or African American*; 4 = *Native Hawaiian or other Pacific native*; and 5 = *European American*), level of education (1 = *less than high school*; 2 = *some high school*; 3 = *high school graduated or GED*; 4 = *some college or vocational school*; and 5 = *college graduated or higher*), marital status (1 = *married*; 2 = *widowed*; 3 = *divorced*; 4 = *separated*; and 5 = *other*), and number of persons in households.

**Chronic Conditions**. Participants self-reported if with a chronic health condition (*yes* or *no*), including arthritis, breathing conditions, depression, diabetes, heart diseases, and vision limitations.

**Personal Perception of Fall and Fall History**. Participants were asked whether (*yes* or *no*) they perceived limitations of social activities due to concerns of potential falls and subsequent injuries. Fall history was measured with the number of falls in the previous three months and the number of falls which resulted in injuries. Fear of falls was measured by a four-point Likert scale ranging from 1 (*Not at all*) to 4 (*A lot*).

**Health Status**. Participants self-rated their present physical functioning health status on a five-point Likert scale ranging from 1 (*Poor*) to 5 (*Excellent*).

**The Confidence in Self-Management of Falls (CSMoF)**. The CSMoF scale was measured on four items on a five-point Likert scale ranging from 1 (*Not at all sure*) to 4 (*Very sure*). Example items of the measure included “I can get up when I fall”, “I can reduce falls”, “I can protect myself while falling”, “I can increase my strength to prevent falls”, and “I can improve my steady”(from AMOB manual, ver. 2013). The total possible score for this scale was 20, with higher total points referring to a higher level of CSMoF. We observed a Cronbach’s alpha of 0.837 for scores from the CSMoF. A principal component analysis was performed (outcome not reported here) with the scale and identified only one factor from this scale, indicating a good measurement validity.

### 2.4. Data Screening

Missing data were inspected by running frequencies. Each variable had missing data for from 6 to 23 cases, with none of the variables having missing cases for over 10% of the total cases. To determine the nature of missing data, the missing cases were coded zero, and non-missing cases were recoded to one. A binary correlational analysis of the recoded variables was then adopted and reported no statistically significant correlation between the recoded variables, indicating that the pattern of missing data is likely to be missing at random (MAR) [30,31]. This means that the missing data in this study were most likely caused by omissions of participants. Thus, mean substitution was made for continuous variables (e.g., age, household, and CSMoF), and mode substitution was applied for dichotomous and ordinal variables (e.g., education, marital status, and health status) [32]. Table 2 displays the descriptive data of variables in the regression analyses.

Statistical analyses were performed by the Statistical Package for the Social Sciences (SPSS, version 24, IBM, Armonk, NY, USA). We utilized general linear regressions to explore the association between CSMoF and target variables. Both simultaneous and hierarchical regressions were used.

**Data analysis**. Three simultaneous regressions were conducted to determine the predictors of the CSMoF from demographics, factors, chronic conditions, and perception of falls. The perception-of fall simultaneous regression utilized predictor variables: (a) limitations; (b) fall frequencies; (c) injury history; (d) fear of falls; and (e) overall health status. CSMoF served as dependent variables for all three regression models. The demographic simultaneous regression was conducted with predictor variables: (a) age; (b) living alone; (c) sex; (d) Hispanic (e) ethnicity; (f) education; (g) marital status; and (h) persons in household. The chronic condition simultaneous regression was conducted with: (a) arthritis; (b) breath issues; (c) depression; (d) diabetes; (e) heart diseases; and (f) vision limitations. The statistically significant predictors from the individual regressions were entered into the final regression analysis to determine the contribution to the association between predictors and CSMoF.

We computed collinearity diagnostics prior to conducting the regression analysis to ensure that the zero-order correlations were below the threshold level of concern (*r* ≤ 0.80), that the variance inflation factors did not exceed 10, and that the tolerance values were close to one [33]. In addition, as checks for the stability of regression coefficients, we applied the sequence of simple slope tests for the regression model, and the rescaled mean-centered values [34]. To counter the probability of Type I errors, the significance value was set at the 95% confidence interval level (*p* ≤ 0.05). For effect size magnitude interpretation, we then computed Cohen’s *f*^2^, which is an index of local effect size (i.e., one variable’s effect size within the context of a multivariate regression model) [31]. We considered parameters for the correlation coefficients as a small effect when *f*^2^ ≥ 0.02, a medium effect when *f*^2^ ≥ 0.15, and a large effect when *f*^2^ ≥ 0.35.

## 3. Results

**Descriptive Statistics**. As shown in Table 1, nearly half (*n* = 359, 52%) of participants reported having arthritis conditions, 94 (13.6%) reported having breathing issues, 80 (11.6%) were diagnosed with depressive symptoms, 113 (16.4%) reported diabetic conditions, 163 (23.6%) had heart diseases, and 98 (14.2%) claimed vision limitations.

Participants showed the least confidence in self-protection, as shown in “I can protect myself while falling” (*M* = 2.32, *SD* = 0.88), followed by “I can get up when I fall” (*M* = 2, *SD* = 0.95), “I can reduce falls” (*M* = 2.81, *SD* = 0.85), and “I can improve my steady” (*M* = 2.95, *SD* = 0.82), and presented the most confidence in “I can increase my strength to prevent falls” (*M* = 3.10, *SD* = 0.82). The total score of CSMoF ranged from 5 to 20 (*M* = 13.99, *SD* = 3.37). The 25th, 50th, and 75th percentiles were 12, 14, and 16, respectively.

**Demographic variables**. The model to predict CSMoF from demographic variables was statistically significant, *R* = 0.178, *R*^2^ = 0.032, *F* = 2.777, *p* = 0.005 (*f*^2^ = 0.031, small effect size). Among the predictor variables, age (β = −0.136, *t* = −3.410, *p* = 0.001) and sex (β = 0.098, *t* = 2.414, *p* = 0.016) were statistically significantly associated with the CSMoF. When holding other demographics constant, every ten years of increase in age would lower the CSMoF by 0.71. Males were more likely to have a higher level of CSMoF by 0.77 points when controlling for other variables.

**Chronic Conditions**. The model to predict CSMoF from chronic health condition was statistically significant with *R* = 0.220, *R*^2^ = 0.048, *F* = 5.808, *p* < 0.001 (*f*^2^ = 0.053, small effect size). Self–reporting with arthritis (β = −0.089, *t* = −2.323, *p* = 0.020), diabetes (β = −0.124, *t* = −3.316, *p* = 0.001), heart disease (β = −0.081, *t* = −2.103, *p* = 0.036), and vision limitations (β = −0.083, *t* = −2.223, *p* = 0.02) were significantly associated with lower CSMoF. Overall, older adults without chronic health conditions reported higher CSMoF levels than those with chronic health conditions.

**Perception of Falls, Physical Limitations, Health Status, Fear of Falls and CSMoF**. This model accounted for statistically significant differences in predicting the CSMoF, *R* = 0.513, *R*^2^ = 0.263, *F* = 48.877, *p* < 0.001 (*f*^2^ = 0.357, large effect size). Physical (function) limitation (β = −0.114, *t* = −3.259, *p* = 0.001), fear of falls (β = −0.365, *t* = −10.405, *p* < 0.001), and overall health status (β = 0.198, *t* = 5.554, *p* < 0.001) significantly predicted CSMoF. Those who self-perceived with physical limitations and fear of falls had lower CSMoF scores, whereas those reporting higher overall health status also had higher level of the CSMoF.

**Figure 1:**
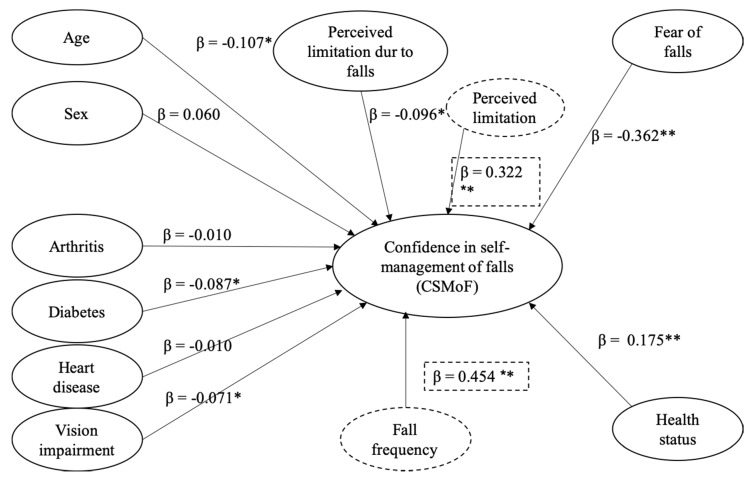
Conceptual model of the regression outcome that demonstrates the relationship between confidence in self-management of falls (CSMoF) and its predictors included in the final model. *Note*. Dashed-line circles and boxes refer to interaction variables. * *p* < 0.05, ** *p* < 0.01.

**Final Model**. Figure 1 shows the conceptual regression model that demonstrates the relationship between confidence in self-management of falls (CSMoF) and its predictors included in the final model. The final simultaneous regression analysis applied the statistically significant predictor variables in the three individual regressions to determine the magnitude of contributions of each predictor variable to the CSMoF. The predictor variables for the final model were: (a) age; (b) sex; (c) arthritis; (d) diabetes; (e) heart diseases; (f) vision limitation; (g) perceived limitations due to falls; (h) overall health status; and (i) fear of falls. The final model was statistically significant, *R* = 0.534, *R*^2^ = 0.286, *F* = 30.237, *p* < 0.001, indicating that 28.6% of the variance in the CSMoF could be explained by the variance in the predictors in the model (*f*^2^ = 0.40, large effect size). The results from these analyses indicate that the fear of falls is strongly associated with the confidence of the CSMoF.

Among all the predictors, age (β = −0.107, *t* = −3.177, *p* = 0.002), diabetes (β = −0.087, *t* = −2.607, *p* = 0.009), vision limitations (β = −0.071, *t* = −2.146, *p* = 0.032), limitations (β = −0.096, *t* = −2.687, *p* = 0.007), overall health status (β = 0.175, *t* = 4.826, *p* < 0.001), and fear of falls (β = −0.362, *t* = −10.264, *p* < 0.001) were still statistically significant. However, sex (β = 0.060, *t* = 1.742, *p* = 0.082), arthritis (β = −0.010, *t* = −0.283, *p* = 0.778), and heart disease (β = −0.010, *t* = −0.286, *p* = 0.775) were no longer statistically significant. The reduced regression coefficient of sex indicated a mediation effect yet was not statistically significant in the later interaction analyses. The significant shrinkage of regression coefficients of arthritis and heart disease on CSMoF when health status was entered the model could be attributed to the variances reported in health status.

Table 3 and Table 4 present the outcome of regression analyses and standardized regression coefficients (*β*) for the predictor variables at each step and in the final model.

We conducted hierarchical regression to determine the impacts of fear of falls while controlling other predictor variables. Fear of falls was introduced into the hierarchical regression at the second step after all other predictor variables tested. The outcome resulted in a statistically significant *R*^2^ change (Δ*R*^2^ = 0.111, *p* < 0.001), indicating that, while controlling all other predictor variables, fear of falls still had the most unique contribution to the CSMoF.

**Interaction effects**. We further analyzed the interaction effects of sex, chronic conditions, fall frequency, health status, and perceived limitation on CSMoF. Table 5 shows the results for the interaction factors for the significant variables from the final model analysis (as reported above).

The interaction term between fear of fall and gender on predicting CSMoF showed non–significant interactive effect, *F*(3687) = −0.176, *p* = 0.078. Similarly, the interaction term between fear of fall and whether or not a participant had chronic conditions was not a significant predictor of CSMoF, *F*(3687) = −0.019, *p* = 0.854; neither was the interaction term between fear of fall and health status a significant of CSMoF, *F*(3687) = −0.108, *p* = 0.414.

**Figure 2:**
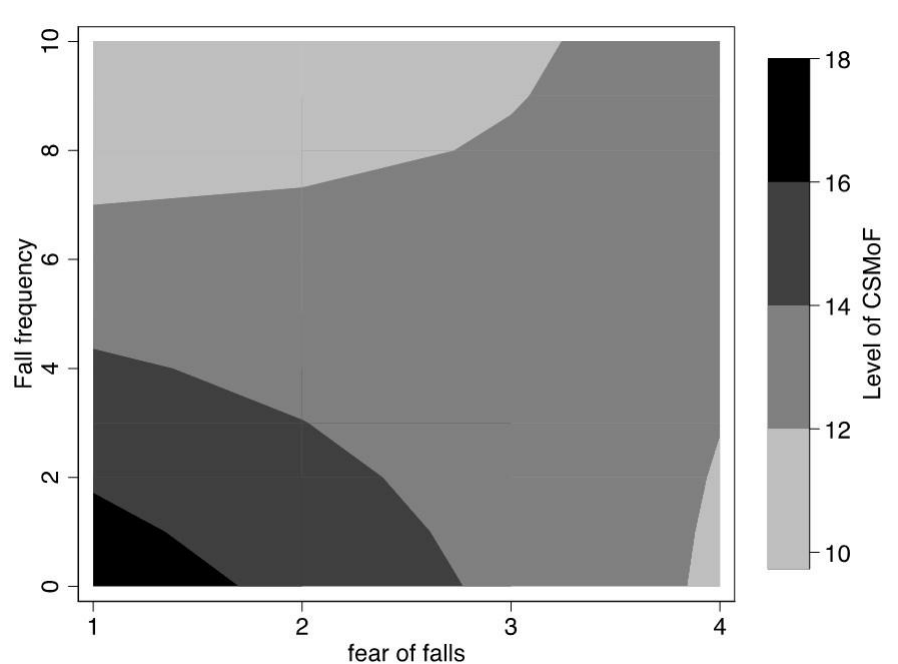
The interaction effect between fall frequency and fear of falls on the level of CSMoF. Note: The darker shaded areas stand for higher levels of CSMoF and lighter areas for lower levels of CSMoF.

Figure 1 presents the results of the analysis for the interaction term effect between fall frequency and fear of falls on CSMoF, which was statistically significant, *F*(3687) = 0.454, *p* = 0.001. Both fall frequency and fear of falls negatively predicted the level of CSMoF. This inverse relationship was particularly true among older adults who self–reported with a history of up to two times and who also reported a fear of falls lower than two out of four (see Figure 2 for a visual representation of the interactional relationship).

**Figure 3:**
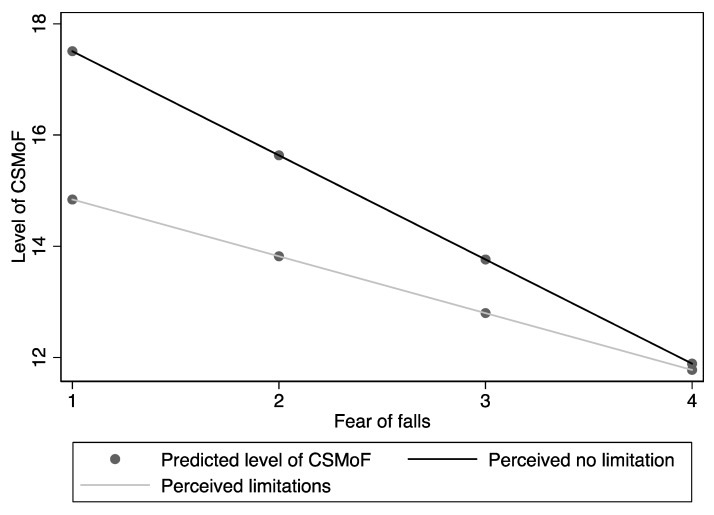
The interaction effect of perceived limitation with fear of falls on level of CSMoF.

Similarly, the interaction term between perceived functional limitation and fear of falls predicted level of CSMoF, *F*(3687) = 0.322, *p* = 0.009 (see Figure 3 for visual representation of the interactional relationship). This means that older adults who self-perceived with no functional limitations and lower levels of fear of falls reported a higher level of CSMoF compared to those who self-perceived with functional limitations. When the fear of falls increases, the level of CSMoF decreases more drastically among older adults who self-perceived limitations due to falls, compared to those who did not.

## 4. Discussion

The subjective, cognition-related personal perceptions of self and falls (i.e., sensations and perceptions) explained the most variance of the level of CSMoF. This is a particularly important finding in view of the fact previous studies premised risk for falls primarily on physical frailty [35,36] rather than cognition related variables, such as self-confidence and risk perception. Consideration of cognition related variables together with physical function capabilities by fall prevention programs for older adults would make for more holistic interventions, resulting in fewer actual falls by seniors.

In addition, older adults who perceived higher physical functioning were less likely concerned about the risk of falls, coming from their confidence in motion controlling. A high level of perceived limitations predicted a lower level of CSMoF [14,15]. Older adults may instead be staying at home rather than being socially active only to avoid potential falls [16]. Programs aimed at building a higher sense CSMoF in older adults should focus on training seniors in recognizing the feasibility of performing certain activities without falling in strategies to perform daily activities of living without falls, and in fear of falls syndrome recovery in those cases when falls do happen. In addition, CSMoF is important to older adults for safety from getting injured due to falls, what to do when falls do happen, including who to call for help, becomes more crucial in establishing CSMoF. We found fear of falls to predict CSMoF over significantly and above demographic and health status variables (large effect size), indicating that the fear of falls may hamper older adults in their in-home and community living participation [20,21].

While older age was associated with lower CSMoF, perhaps from declining physical functioning [37], individual older adults may have different fall risk outcomes from their physical activities self-efficacy, minimizing their risk for falls [25,38]. The tendency to treat older adults, age 65 and over, as a homogeneous group in fall prevention programming could be a mistake. Program designers aiming to improve the CSMoF should individualize interventions to participants, adapting or customizing sessions and timeframes based on physical, cognitive, and mental conditions.

In line with previous studies, having chronic conditions was associated with lower levels of CSMoF, particularly when having either diabetes or vision limitations or both. It is probable that older adults with diabetes tend to have weaker lower-limb strength and limited mobility due to vitamin D deficiency [39]. Likewise, vision limitations from the aging process, such as loss of contrast sensitivity, depth perception, visual field, and visual motion perception, may strongly impede older adults’ CSMoF [40]. Visual loss also occurs with uncontrolled diabetes, which speaks to the importance of physical functioning to CSMoF among older adults.

In summary, findings of this study have the three main implications for fall prevention design: (1) inclusion of CSMoF in fall prevention programs; (2) modular design where each session can be modified to accommodate older adults’ subjective personal factors; and (3) integrative programs that address disability, health and functioning holistically [41]. Fall prevention programs customized to older adults’ confidence in self-managing falls could benefit older adults with a variety of physical functional statuses, including those who may be taking multi-pharmacy regimens.

This study has some limitations. Firstly, this was a cross-sectional study that sampled mostly well-educated older white Americans. For that reason, findings may not generalize to the population of older adult Americans or those from other settings. Secondly, the measurement of health status and chronic conditions were dichotomous rather than on continuous or ordinal scales, which may bias the statistical power when using multiple regression. Single item measures may misrepresent self-reporting of health statuses or conditions [42,43]. Third, we analyzed by chronic conditions reported by the area agency on their older adult clients and with the possibility that an unknown number of older adults may have been with comorbid conditions influencing their fall risk self-confidence management. Fourth, the measurement of fear of fall in this study was, again, chosen by the agency. Future studies should utilize a longitudinal design and multiple-item measures with a more ethnically diverse sample of older adults for greater confidence in the findings.

## 5. Conclusions

Fear of falls was the strongest predictor of CSMoF development. Moreover, older adults who were from different age cohorts, diagnosed with diabetes, had vision limitations, and perceived higher levels of limitations due to falls and fear of falls were more likely to have low CSMoF. Falls can be very dangerous for older adults if not properly managed. This study found evidence for personal demographics, history of chronic illness, and prior falls with injury to predict CSMoF. Educational interventions targeting the improvement of CSMoF among older adults should take into account the demographics and personal histories of seniors, including chronic condition diagnoses, perceived limitations due to falls, and the fear of falls.

## Figures and Tables

**Table 1:** Participant characteristics (N = 691).

Characteristics	*n* (%)	*M* (*SD*)
**Demographic variables**		
Age		76.23 (6.44)
Living alone		
Yes	229 (33.1)	
No	462 (66.9)	
Sex		
Male	165 (23.9)	
Female	526 (76.1)	
Hispanic		
Yes	17 (2.5)	
No	674 (97.5)	
Ethnicity		
American Indian or Alaska native	4 (0.6)	
Asian American	20 (2.9)	
Black or African American	10 (1.4)	
Native Hawaiian or other Pacific native	3 (0.4)	
European American	654 (94.7)	
Education		
Less than high school	2 (0.3)	
Some high school	4 (0.6)	
High school graduated or GED	67 (9.7)	
Some college or vocational school	220 (31.8)	
College graduated or higher	398 (57.6)	
Marital status		
Married	400 (57.9)	
Widowed	190 (27.5)	
Divorced	70 (10.1)	
Separated	3 (0.4)	
Other	28 (4.1)	
Persons in household		
1	229 (33.1)	
2	426 (61.6)	
3	20 (2.9)	
4	4 (0.6)	
5	2 (0.3)	
6	1 (0.1)	
Did not report	9 (1.3)	

**Table 2:** Data of Variables in the regression analysis.

Characteristics	*n* (%)	*M* (*SD*)
**Chronic conditions**		
Arthritis		
Yes	359 (52)	
No	332 (48)	
Breathing		
Yes	94 (13.6)	
No	597 (86.4)	
Depress		
Yes	80 (11.6)	
No	611 (88.4)	
Diabetes		
Yes	113 (16.4)	
No	578 (83.6)	
Heart disease		
Yes	163 (23.6)	
No	528 (76.4)	
Vision limitations		
Yes	98 (14.2)	
No	593 (85.8)	
**Perception of falls**		
Limitation		
Yes	212 (30.7)	
No	479 (69.3)	
Fall frequency		0.51 (1.22)
Resulted in injury		0.16 (0.58)
Fear of falls		2.86 (0.87)
Health status		3.34 (0.80)
Poor	1 (0.001)	
Fair	88 (12.8)	
Good	326 (47.2)	
Very good	226 (32.7)	
Excellent	50 (7.2)	

**Table 3:** Outcome of three separate regressions for predictor selection (N = 691).

	*R* ^2^	*B*	*SE B*	β
**Demographic variables**	0.032 *			
Age		−0.071	0.021	−0.136 *
Living alone		0.292	0.342	0.041
Sex		0.773	0.32	0.098*
Hispanic		0.398	0.825	0.018
Ethnicity		−0.088	0.204	−0.016
Education		0.138	0.182	0.029
Marital status		−0.139	0.125	−0.046
Persons in household		0.225	0.169	0.057
**Chronic conditions**	0.048 **			
Arthritis		−0.597	0.257	−0.089 *
Breathing		−0.28	0.379	−0.028
Depress		−0.489	0.401	−0.046
Diabetes		−1.131	0.341	−0.124 *
Heart disease		−0.645	0.307	−0.081 *
Vision		−0.803	0.361	−0.083 *
**Perception of falls**	0.263 **			
Limitation		−0.831	0.255	−0.114 *
Fall frequency		−0.098	0.107	−0.035
Resulted in injury		0.179	0.223	0.031
Fear of falls		−1.419	0.136	−0.365 **
Health status		0.836	0.151	0.198 **

*Note*. Model for demographic variables: *R* = 0.178, *R*^2^ = 0.032, *F* = 2.777, *p* = 0.005; Model for chronic conditions: *R* = 0.220, *R*^2^ = 0.048, *F* = 5.808, *p* < 0.001; Model for perception of falls: *R* = 0.513, *R* = 0.263, *F* = 48.877, *p* < 0.001. * *p* < 0.05, ** *p* < 0.01.

**Table 4:** The Outcome of the final simultaneous regression analysis (N = 691).

	*R* ^2^	*B* (95% CI)	*SE B*	β
	0.286 **			
**Demographic variables**				
Age		−0.056 (−0.091, −0.021)	0.018	−0.107 *
Sex		0.478 (−0.061, 1.016)	0.274	0.060
**Chronic conditions**				
Arthritis		−0.066 (−0.521, 0.390)	0.232	−0.010
Diabetes		−0.797 (−1.397, −0.197)	0.306	−0.087 *
Heart disease		−0.077 (−0.604, 0.451)	0.269	−0.010
Vision		−0.682 (−1.305, −0.058)	0.318	−0.071 *
**Perception of falls**				
Limitation		−0.702 (−1.215, −0.189)	0.261	−0.096 *
Health status		0.743 (0.441, 1.045)	0.154	0.175 **
Fear of falls		−1.404 (−1.673, −1.136)	0.137	−0.362 **

*Note*. The final model: *R* = 0.534, *R*^2^ = 0.286, *F* = 30.237, *p* < 0.001. * *p* < 0.05, ** *p* < 0.01.

**Table 5:** Results from interaction analyses.

Variable Interacted with Fear of Fall in Prediction of CSMoF	β
Sex	−0.176
No chronic condition	−0.019
fall frequency	0.454 **
Health status	−0.108
Perceived limitation	0.322 **

*Note*. For each pair of analysis, the degree of freedom was 3687. * *p* < 0.05, ** *p* < 0.01.
